# Quantitative three-dimensional imaging of *Coxiella burnetii* infection by focused ion beam-scanning electron microscopy

**DOI:** 10.1128/iai.00007-26

**Published:** 2026-04-29

**Authors:** Jack M. Botting, Samuel Steiner, Morven Graham, Xinran Liu, Craig R. Roy, Jun Liu

**Affiliations:** 1 Microbial Sciences Institute, Yale University 5755, West Haven, Connecticut, USA; 2 Department of Microbial Pathogenesis, Yale School of Medicine 12228 , New Haven, Connecticut, USA; 3 Center for Cellular and Molecular Imaging, Electron Microscopy Core Facility, Yale School of Medicine 12228 , New Haven, Connecticut, USA; University of Pennsylvania Perelman School of Medicine, Philadelphia, Pennsylvania, USA

**Keywords:** *Coxiella*-containing vacuole, secretion system, FIB-milling, intracellular pathogen, volume EM

## Abstract

*Coxiella burnetii* is a highly virulent intracellular pathogen and the causative agent of acute and chronic Q fever in humans. Central to its pathogenicity is the Dot/Icm type IV secretion system, which translocates more than 100 effector proteins into host cells to drive the biogenesis of *Coxiella*-containing vacuoles (CCVs), which are specialized, lysosome-derived compartments that support bacterial replication. During intracellular growth, *C. burnetii* undergoes a distinctive biphasic developmental cycle, transitioning between the infectious, dormant small cell variant and the replicative large cell variant. Although this developmental transition is tightly linked to CCV biogenesis, the underlying mechanisms remain poorly understood. Here, we combine advanced focused ion beam-scanning electron microscopy (FIB-SEM) with machine learning-based data analysis to visualize *C. burnetii*-infected host cells in three dimensions. This approach reveals striking pleiomorphism in both bacterial morphology and CCV architecture. Moreover, we demonstrate that FIB-SEM-based quantitative analysis can sensitively detect CCV biogenesis defects in *C. burnetii* mutants that fail to generate the large vacuoles characteristic of wild-type infection. Specifically, analysis of HeLa cells infected with a *cig2::*Tn mutant confirms that this strain forms smaller CCVs with pronounced defects in homotypic fusion. Our study provides unprecedented three-dimensional insights into the complex intracellular lifestyle of *C. burnetii* and establishes FIB-SEM as a powerful platform for dissecting CCV biogenesis, bacterial development, and mutant phenotypes in infected host cells.

## INTRODUCTION

The gram-negative pathogen *Coxiella burnetii* is the causative agent of Q fever, a zoonotic disease transmitted from livestock to humans that typically presents with flu-like symptoms but can progress to endocarditis and chronic, persistent infection (1). *C. burnetii* is exceptionally infectious, with aerosol transmission occurring at an infectious dose as low as ~10 bacterial cells and further facilitated by the remarkable environmental stability of the organism (2–4). Following inhalation, *C. burnetii* primarily infects alveolar macrophages, where it replicates within a modified phagolysosomal compartment known as the *Coxiella*-containing vacuole (CCV) (5). Although *C. burnetii* is an obligate intracellular pathogen, it can be cultivated axenically in liquid medium and on solid agar, enabling powerful genetic, biochemical, and cell biological approaches to dissect the molecular mechanisms underlying its pathogenicity (6).

The exceptional environmental stability and infectivity of *C. burnetii* are attributed to its unusual biphasic developmental cycle (4). During this cycle, *C. burnetii* alternates between two morphologically and physiologically distinct forms: a dormant small cell variant (SCV) and a replicative large cell variant (LCV). Both variants are straight rods; however, the SCV is markedly shorter than the LCV (0.2–0.5 μm vs ~1 µm, respectively) and contains substantially more condensed genetic material (4, 7). In addition, the SCV cell envelope is characterized by extensive membrane invaginations that project into the cytoplasm. These structures are thought to function as a membrane reservoir that facilitates the rapid expansion of the cell envelope during the transition from SCV to LCV (7).

The SCV represents the infectious form of *C. burnetii* and differentiates into the LCV once the bacterium establishes a permissive intracellular niche. Specifically, SCV-to-LCV differentiation is triggered by acidification of the CCV as it matures along the endocytic pathway and undergoes fusion with lysosomes. This acidification also serves as a key signal for activation of the *C. burnetii* Dot/Icm type IV secretion system (T4SS), which is essential for CCV biogenesis and intracellular replication (5, 7–12).

The primary function of the *C. burnetii* Dot/Icm T4SS and its translocated effector proteins is to remodel the CCV into a compartment that supports bacterial replication. For example, the effectors Cig2 and Cig57 play critical roles in generating a replication-permissive CCV and in maintaining the vacuole in an autolysosomal state by promoting fusion of autophagosomes with the CCV (13–18). Consistent with these observations, T4SS activity is essential for intracellular replication and CCV biogenesis (8, 14, 19, 20). Fusion of CCVs with autophagosomes, as well as constitutive homotypic fusion of early phagocytic vesicles containing *C. burnetii*, is mediated by host factors, including SNARE proteins and components of the autophagy machinery (14, 15, 21, 22). Three-dimensional imaging approaches capable of directly visualizing these *C. burnetii*-host interactions during infection are therefore crucial for dissecting the mechanistic contributions of bacterial effectors and host proteins to CCV biogenesis.

Among electron microscopy (EM) modalities capable of probing pathogen-host interactions at nanometer resolution, focused ion beam-scanning electron microscopy (FIB-SEM) has emerged as a powerful approach, offering high isotropic resolution (<10 nm in *x*, *y*, and *z*) and fully automated volume acquisition (23). These features are particularly advantageous for quantitative three-dimensional analysis of complex intracellular environments. Here, we apply FIB-SEM to image *C. burnetii* populations within infected host cells and develop machine learning-guided analysis pipelines to systematically segment and quantify bacteria, CCVs, and host cell features. This integrative approach yields extensive quantitative information on the spatial organization and morphological heterogeneity of *C. burnetii* infection. Furthermore, comparison of cells infected with wild-type bacteria and a *cig2::*Tn mutant—defective in CCV biogenesis and homotypic fusion of lysosome-derived vesicles—reveals pronounced differences in the three-dimensional pleiomorphism of both bacteria and CCVs. Taken together, our findings establish FIB-SEM as a robust platform for quantitative analysis of *C. burnetii* infection and open new avenues for investigating host-pathogen interactions across diverse intracellular pathogens.

## RESULTS

### FIB-SEM enables quantitative three-dimensional visualization of *C. burnetii* infection

**Fig 1:**
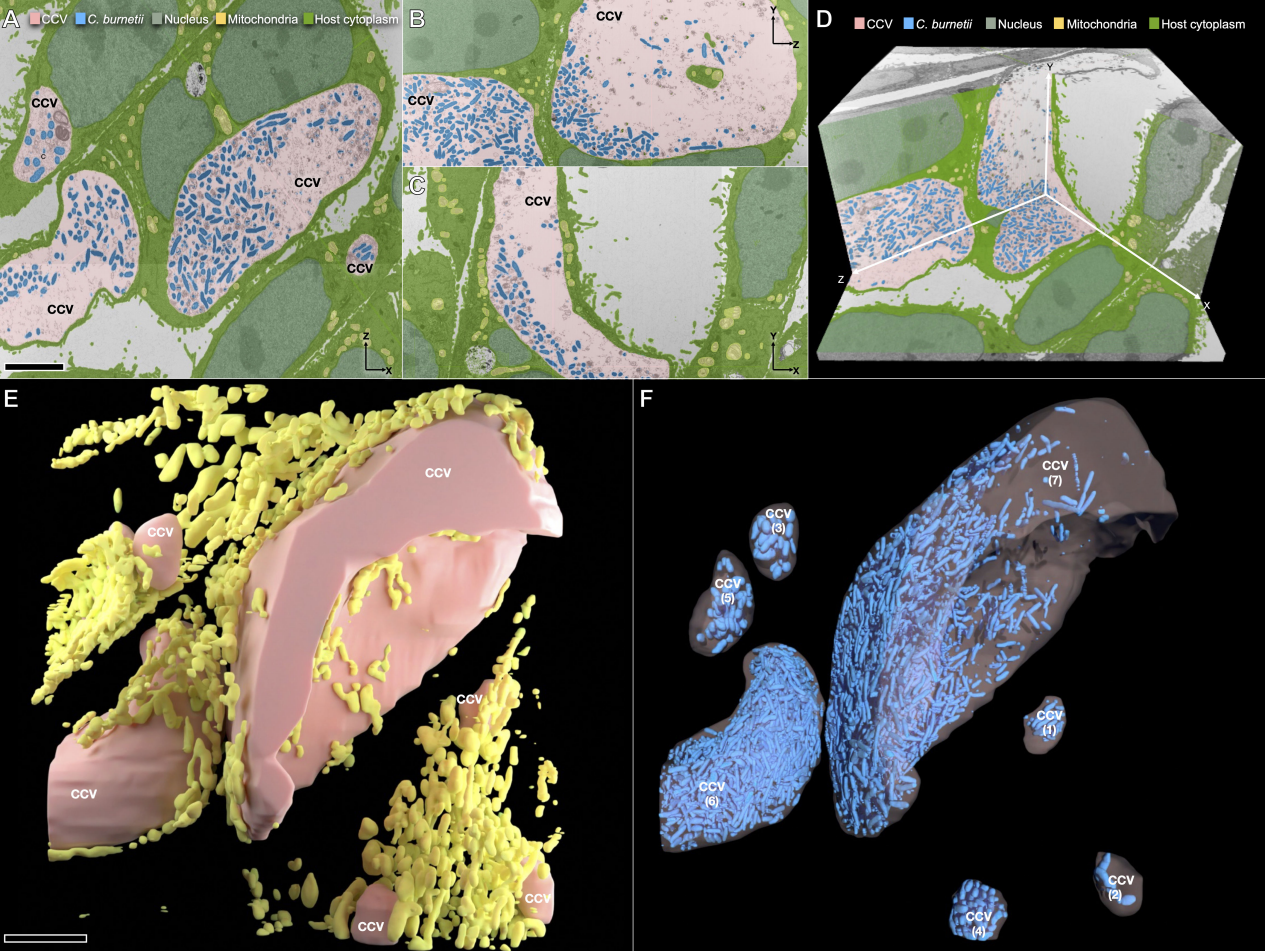
FIB-SEM enables three-dimensional visualization of HeLa cells persistently infected with wild-type *C. burnetii*. (**A–C**) Representative segmented slices from the FIB-SEM data set showing HeLa cells persistently infected with wild-type *C. burnetii*. Host cytoplasm (bright green), nuclei (dark green), mitochondria (yellow), *C. burnetii* cells (blue), and CCVs (pink) are indicated. Scale bar, 5 μm. (**D**) Three orthogonal slices from the three-dimensional FIB-SEM data set. (**E**) Three-dimensional rendering of CCVs and mitochondria present in the data set. Scale bar, 5 μm. (**F**) Same three-dimensional rendering as in panel (**E**), with CCV membranes rendered transparent to reveal *C. burnetii* cells within the vacuoles. CCVs are numbered in order of increasing volume.

**Fig 2:**
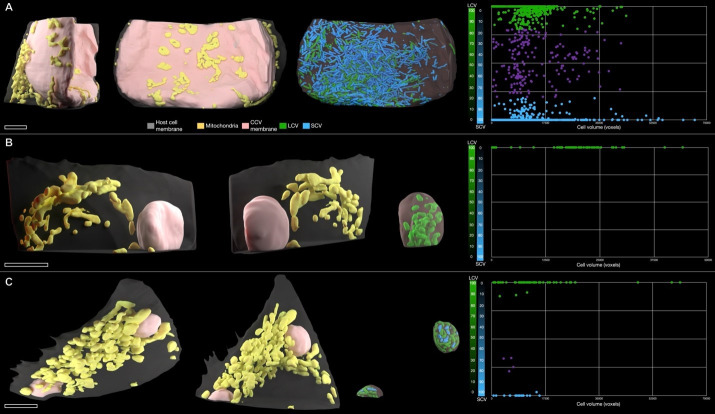
CCV morphology is highly diverse in persistently infected HeLa cells. (**A**) Three-dimensional rendering of a HeLa cell containing a single large CCV and voxel-by-voxel segmentation of intracellular *C. burnetii*. CCV (pink), mitochondria (yellow), *C. burnetii* SCVs (blue), and LCVs (green) are shown. Right, scatter plot of individual *C. burnetii* cells within the HeLa cell shown in (**A**), relating cell volume to variant classification. LCVs (green), intermediate cells (purple), and SCVs (blue) are indicated. Scale bar, 5 μm. (**B**) Three-dimensional rendering of a HeLa cell containing a smaller CCV with corresponding voxel-by-voxel bacterial segmentation. Right, scatter plot of *C. burnetii* cell volumes versus variant classification for the cell shown in (**B**). Scale bar, 5 μm. (**C**) Three-dimensional rendering of a HeLa cell containing two small CCVs with corresponding voxel-by-voxel bacterial segmentation. Right, scatter plot of *C. burnetii* cell volumes versus variant classification for the cell shown in (**C**). See main text for details of classification criteria for SCV, LCV, and intermediate phenotypes. Scale bar, 5 μm.

We previously used cryo-electron tomography (cryo-ET) to visualize individual *C. burnetii* cells within CCVs and to capture bacterial developmental transitions during infection (7). However, cryo-ET is inherently limited to thin samples and provides restricted volumetric information along the *z*-axis. To visualize large *C. burnetii* populations and the CCVs within intact host cells, we employed FIB-SEM to generate three-dimensional volumes of HeLa cells that were persistently infected with wild-type *C. burnetii* Nine Mile phase II (NMII) and maintained in continuous culture prior to imaging. FIB-SEM data immediately revealed striking heterogeneity in CCV size and morphology. CCV volumes ranged from ~8 µm³ to more than 2,000 μm³ and, in some cases, occupied up to ~60% of the total imaged volume of the host cell (Fig. 1 , 2 and Table 1). Consistent with previous observations for a wild-type *C. burnetii* infection, 5 of the 18 cells analyzed contained a single, large CCV. Notably, one cell contained two small but spatially distinct CCVs (Fig. 2), highlighting variability in CCV organization even under persistent infection conditions.

**TABLE 1:** Statistics of the persistent infection of wild-type *C. burnetii*

CCV	SCV	LCV	Intermediate	Total cells	Volume (µm^3^)
1	4	19	2	25	8.2
2	0	7	0	7	19
3	3	21	0	24	19.2
4	14	37	2	53	21.8
5	0	61	0	61	77.2
6	1,038	196	154	1,388	594.7
7	1,405	464	190	2,059	2,072.7
Average	352	115	49.7	516.7	401.8
Total	2,464	805	348	3,617	2,912.9

In addition to CCVs and bacteria, other major cellular organelles including nuclei and mitochondria readily resolved throughout the volumes (Fig. 1), providing important spatial context for host-pathogen interactions. Acquired at 12 nm isotropic voxel resolution, these data sets capture a three-dimensional snapshot of *C. burnetii* infection with a singular combination of cellular-scale coverage with nanometer-scale resolution.

### Both *C. burnetii* cells and CCVs are highly pleiomorphic

To quantitatively characterize *C. burnetii* populations within CCVs, we implemented automated segmentation of the entire FIB-SEM data set using a convolutional neural network (CNN) in the Dragonfly software package. Following initial manual segmentation to generate ground truth for training and validation, the model was trained on FIB-SEM slices annotated into multiple classes, including *C. burnetii* cells, CCVs, and intravacuolar milieu, cellular debris within CCVs, host cell mitochondria, host cell nuclei, and the remainder of the host cell cytoplasm. After thorough evaluation, the trained model was applied to the full data set, enabling identification and quantitative characterization of 3,617 individual bacterial cells across seven CCVs (Table 1; Movie S1).

To further resolve bacterial developmental states, we trained a second CNN to distinguish SCVs from LCVs. Ground truth for this classifier was generated by manually labeling 250 previously segmented bacterial cells as SCV or LCV based on their characteristic internal density patterns, reflecting the condensed genomes of SCVs relative to LCVs in electron micrographs (Fig. S1) (4). This model produced voxel-level classifications for all bacteria in the data set. Notably, many cells were not classified exclusively as SCV or LCV; instead, different regions of the same cell were assigned distinct variant identities. This observation provided an opportunity to systematically examine intermediate phenotypes previously reported for *C. burnetii* (7). We therefore defined SCVs as cells in which >80% of voxels were classified as SCV (<20% LCV), LCVs as cells with >80% LCV voxels (<20% SCV), and intermediate cells as those falling between these thresholds. Importantly, model predictions did not simply reflect average electron density, as mean cellular density showed no correlation with variant classification (Fig. S1D).

**Fig 3:**
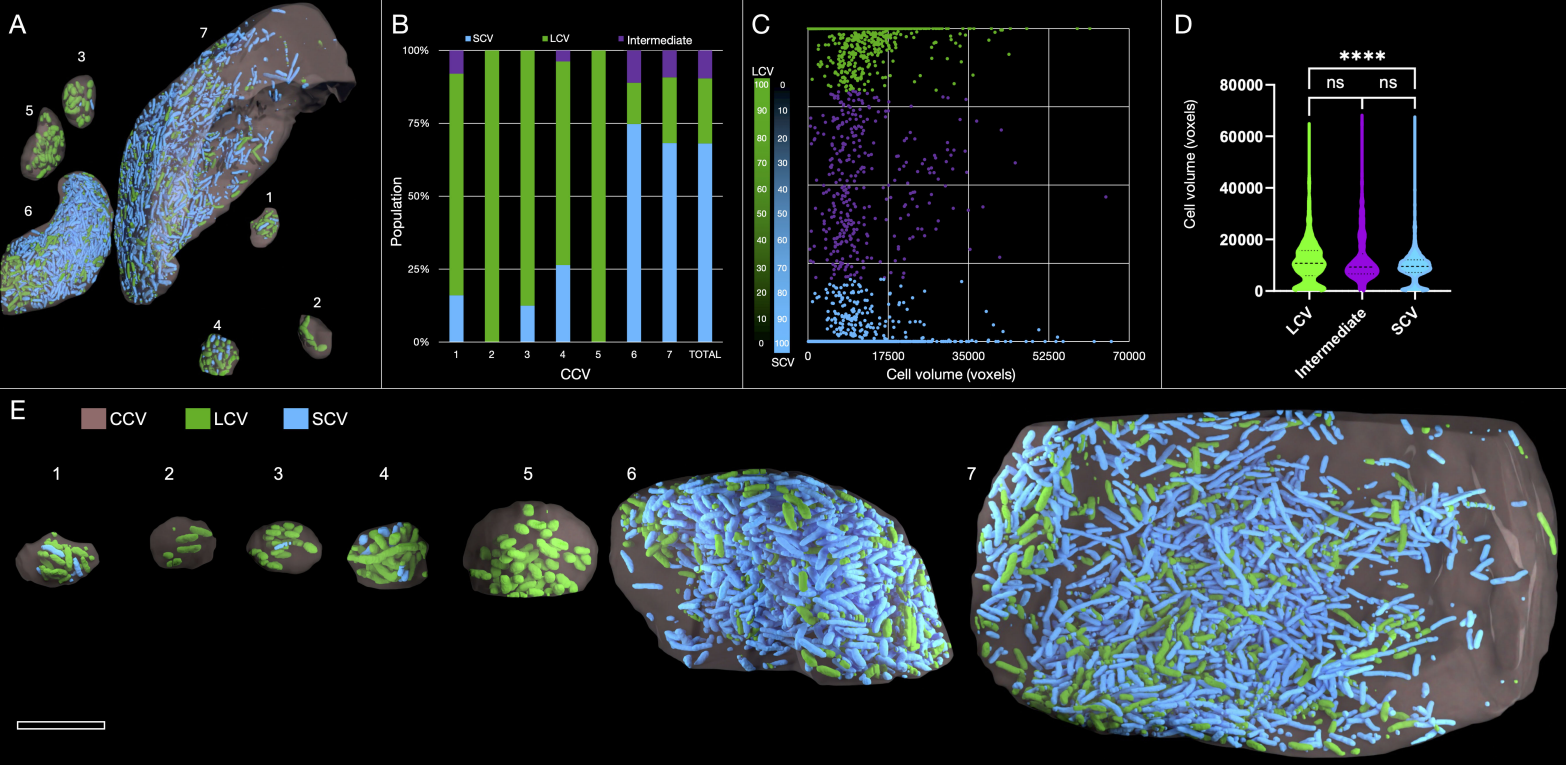
Distribution of bacterial cell variants and cell volumes varies among CCVs. (**A**) Three-dimensional rendering of CCVs present in the wild-type persistent infection FIB-SEM data set, showing voxel-by-voxel segmentation of *C. burnetii* cells within each vacuole. CCVs are numbered in order of increasing volume. (**B**) Bar graph showing the proportion of SCVs, LCVs, and intermediate cells within each CCV. See main text for details of variant classification criteria. (**C**) Scatter plot of individual *C. burnetii* cells relating cell volume to variant classification. LCVs (green), intermediate cells (purple), and SCVs (blue) are indicated. (**D**) Violin plot showing the distribution of cell volumes for each variant population. Statistical significance was assessed using the Mann-Whitney *U* test. (**E**) Lineup of CCVs illustrating voxel-by-voxel segmentation of *C. burnetii* cells within each vacuole. CCVs (pink), SCVs (blue), and LCVs (green) are indicated. Scale bar, 5 μm.

The distribution of bacterial variants and cell volumes varied markedly among CCVs (Fig. 2 and Table 1). Five of the seven CCVs were predominantly or exclusively occupied by LCVs, whereas the two largest CCVs contained populations composed of more than 65% SCVs. Cells classified as intermediate cells comprised no more than 11% of the population in any CCV (Fig. 3A and B), suggesting that these vacuoles represent distinct stages along the infection time course. Cell volumes for SCVs, LCVs, and intermediate cells largely clustered below 20,000 voxels (~0.034 µm³). Notably, some of the largest *C. burnetii* cells in the data set were classified as SCVs (Fig. 3C). Although SCV and LCV volumes overlapped substantially, their distributions were significantly different, as assessed by a Mann-Whitney *U* test (*P* < 0.0001) (Fig. 3D).

**Fig 4:**
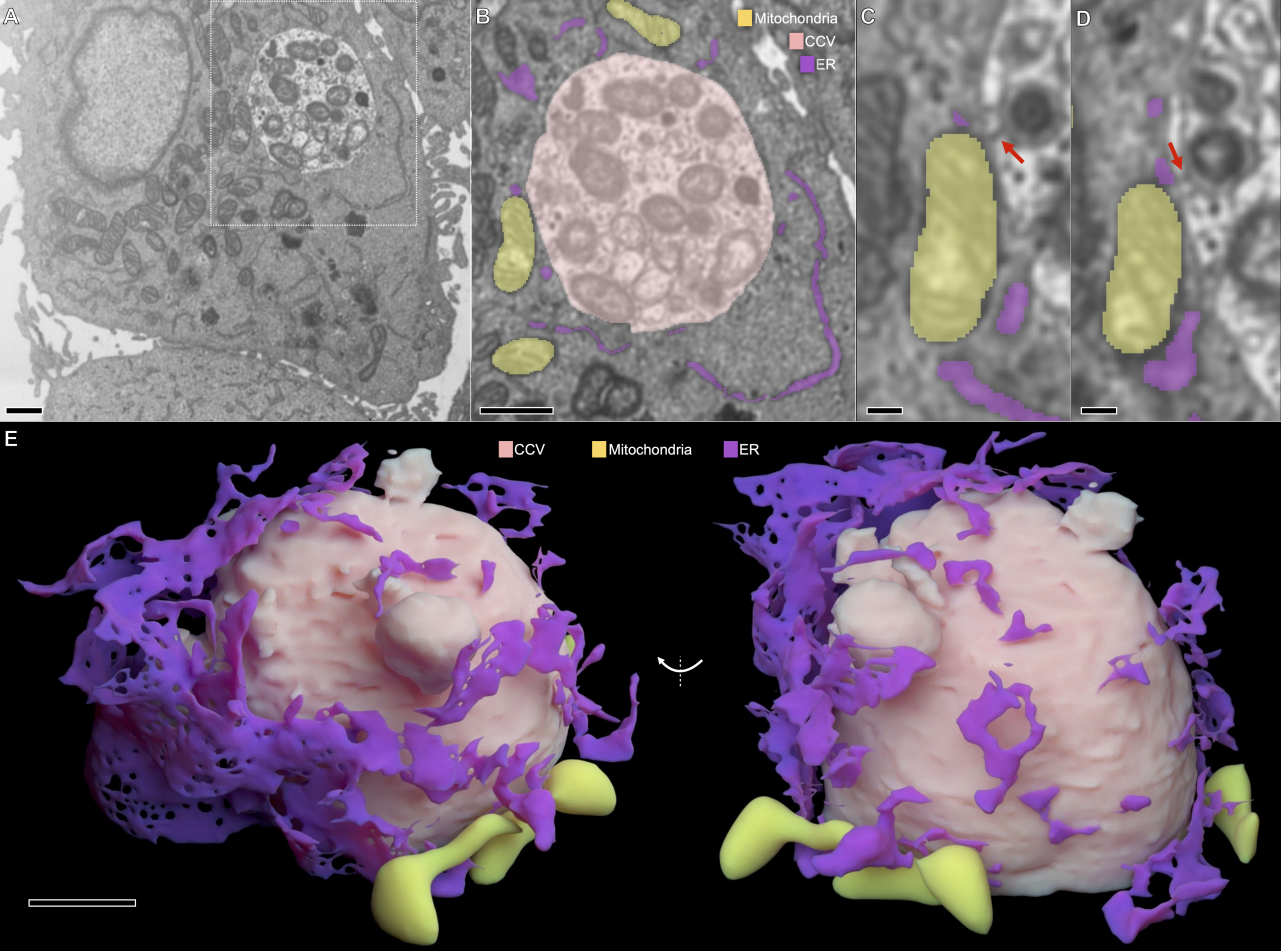
Host cell organelles form few contact sites with the CCV. (**A**) Representative slice from the wild-type persistent infection FIB-SEM data set showing a host cell containing a CCV. Scale bar, 1 μm. (**B**) Pseudocolored segmentation of the CCV shown in (**A**), highlighting endoplasmic reticulum (ER; purple), mitochondria (yellow), and the CCV (pink). Scale bar, 1 μm. (**C and D**) Higher-magnification views of the CCV showing a *C. burnetii* cell connected to a host mitochondrion (**C**) and to the host ER network (**D**). Putative tethering structures are indicated by red arrows. Scale bar, 0.2 μm. (**E**) Three-dimensional renderings of the CCV and associated host organelles shown in panels (**A–D**). Scale bar, 1 μm.

Beyond bacterial and CCV segmentation, we also segmented host cell mitochondria and endoplasmic reticulum (ER) within a large subvolume to examine potential CCV-organelle interactions (Fig. 4). While contacts between CCVs and host organelles were generally sparse, we identified two short, linear densities apparently linking a *C. burnetii* cell to a mitochondrion (~180 nm) and to the ER (~150 nm) (Fig. 4C and D).

Taken together, these machine learning-guided analyses enable robust quantification of multiple highly variable parameters of *C. burnetii* infection, revealing extensive pleiomorphism in both bacterial cells and CCVs and highlighting the power of FIB-SEM for population-level, three-dimensional analysis of intracellular pathogens.

### *C. burnetii* pleiomorphism is observed at 8 days post-infection (dpi)

**Fig 5:**
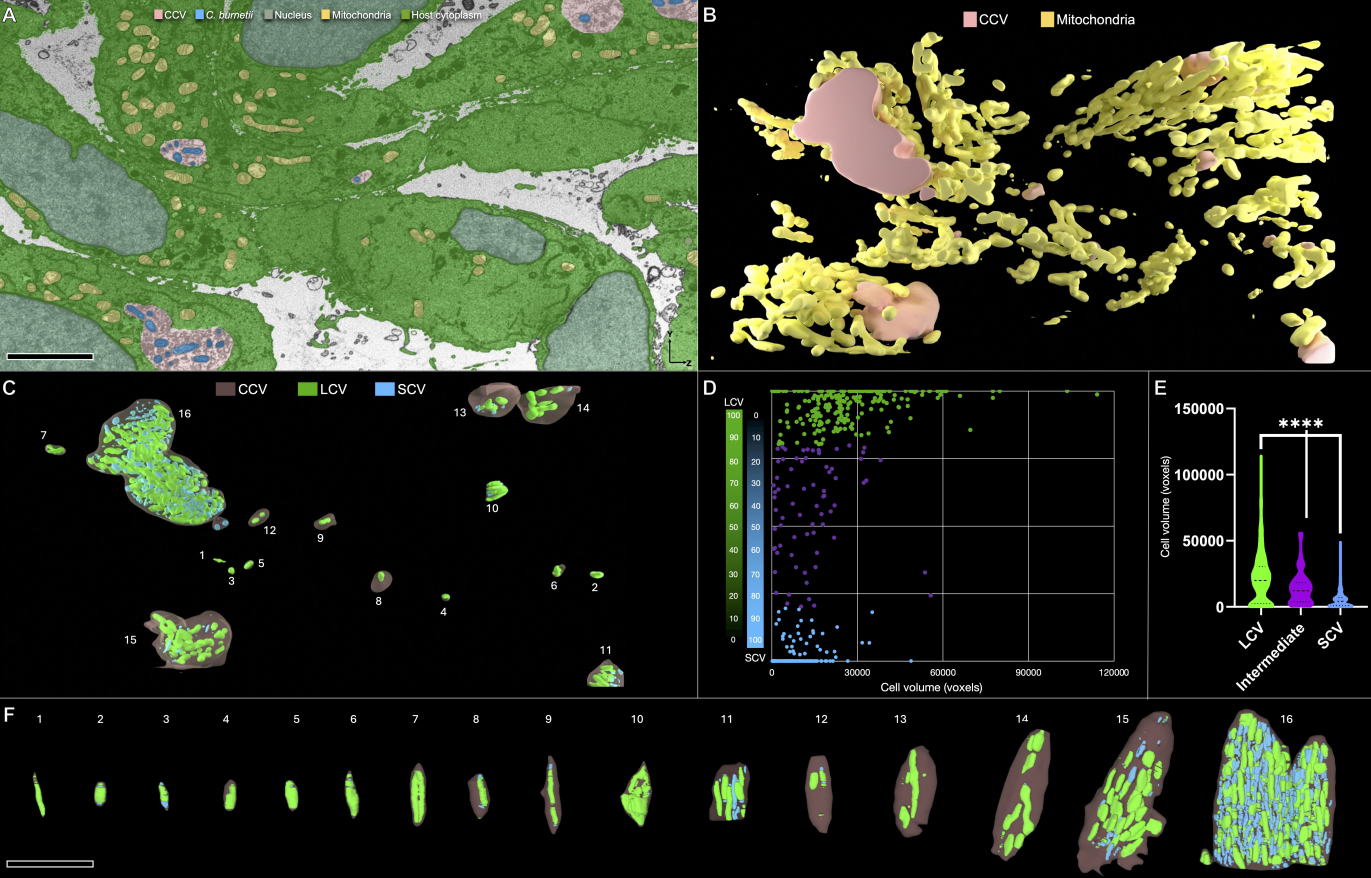
Most CCVs formed by the *cig2::*Tn mutant contain few bacteria at 8 dpi. (**A**) Pseudocolored slice from the *cig2::*Tn mutant FIB-SEM data set acquired at 8 dpi. Host cytoplasm (bright green), nuclei (dark green), mitochondria (yellow), *C. burnetii* cells (blue), and CCVs (pink) are shown. Scale bar, 5 μm. (**B**) Three-dimensional rendering of CCVs and mitochondria present in the data set. (**C**) Three-dimensional rendering of CCVs showing voxel-by-voxel segmentation of *C. burnetii* cells within each vacuole. CCVs are numbered in order of increasing volume. Cells are colored by variant: SCVs (blue) and LCVs (green). (**D**) Scatter plot of individual *C. burnetii* cells relating cell volume to variant classification. LCVs (green), intermediate cells (purple), and SCVs (blue) are indicated. See main text for details of SCV, LCV, and intermediate cell classification criteria. (**E**) Violin plot showing the distribution of cell volumes for each variant population. Statistical significance was assessed using the Mann-Whitney *U* test. (**F**) Lineup of CCVs illustrating voxel-by-voxel segmentation of *C. burnetii* cells within each vacuole. Scale bar, 5 μm.

**Fig 6:**
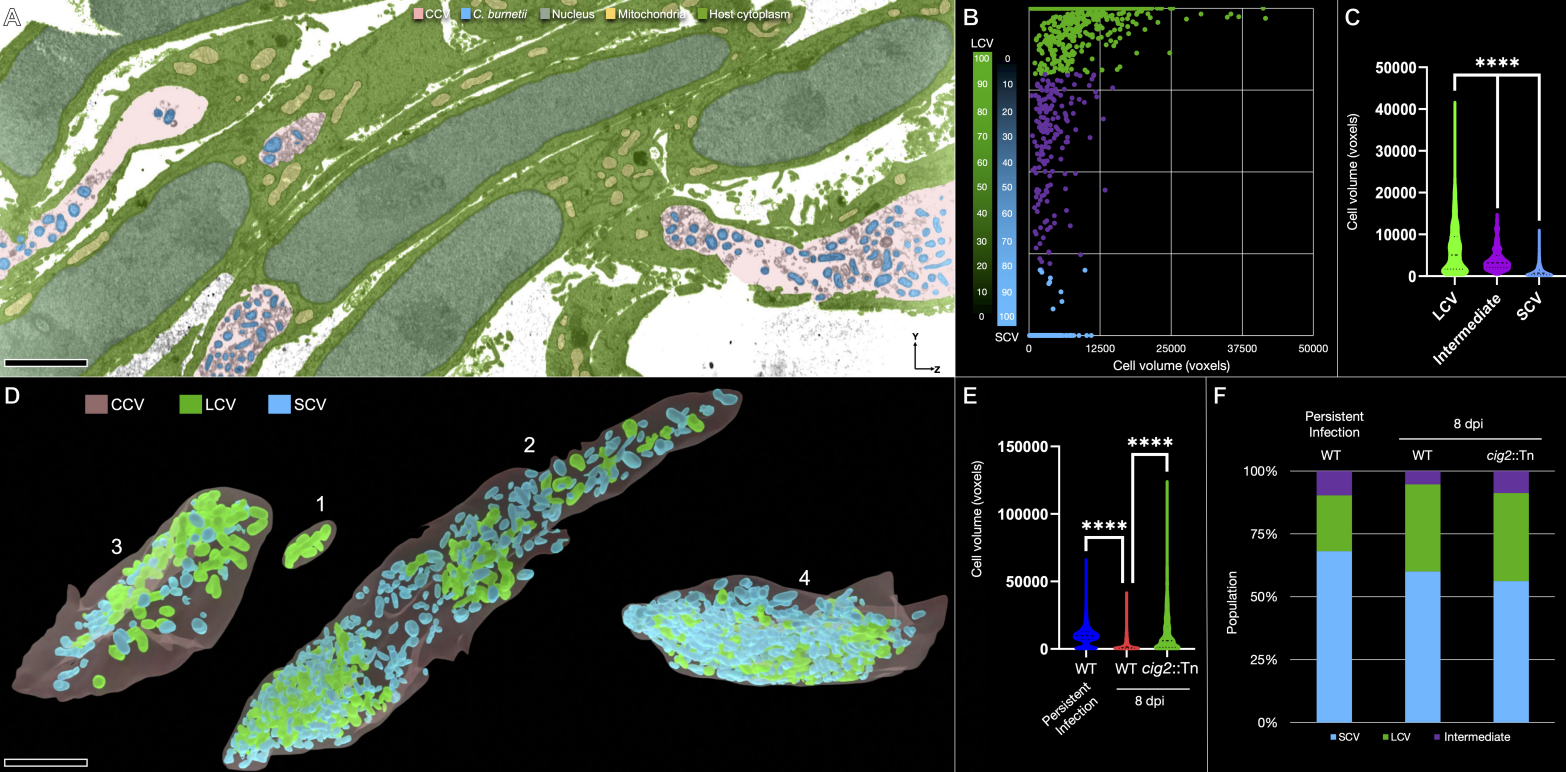
Wild-type *C. burnetii* cells at 8 dpi are smaller than in persistently infected cells. (**A**) Pseudocolored slice from the 8 dpi wild-type FIB-SEM data set. Host cytoplasm (bright green), nuclei (dark green), mitochondria (yellow), *C. burnetii* cells (blue), and CCVs (pink) are shown. Scale bar, 5 μm. (**B**) Scatter plot of individual *C. burnetii* cells relating cell volume to variant classification. LCVs (green), intermediate cells (purple), and SCVs (blue) are indicated. See main text for details of SCV, LCV, and intermediate cell classification criteria. (**C**) Violin plot showing the distribution of cell volumes for each variant population. Statistical significance was assessed using the Mann-Whitney *U* test. (**D**) Three-dimensional rendering of CCVs showing voxel-by-voxel segmentation of *C. burnetii* cells within each vacuole. CCVs are numbered in order of increasing volume. Cells are colored by variant: SCVs (blue) and LCVs (green). Scale bar, 5 μm. (**E**) Violin plot comparing the distributions of bacterial cell volumes across all FIB-SEM data sets. Statistical significance was assessed using the Mann-Whitney *U* test. (**F**) Bar graph showing the proportion of SCVs, LCVs, and intermediate cells in each FIB-SEM data set.

We next applied our FIB-SEM and machine learning-based analysis pipeline to characterize infection by a *C. burnetii cig2::*Tn mutant defective in CCV biogenesis. Cig2 is a Dot/Icm T4SS effector that promotes constitutive fusion between the CCV and autophagosomes, driving the formation of an autolysosomal vacuole with the characteristic fusogenic properties of wild-type CCVs. Loss of Cig2 function results in impaired homotypic fusion between CCVs, leading to a multivacuolar phenotype, reduced CCV size, and attenuated virulence in animal models (14–16). FIB-SEM data sets of HeLa cells infected with wild-type *C. burnetii* or the *cig2::*Tn mutant were acquired at 8 dpi (Fig. 5 and 6).

Consistent with previous reports, multiple CCVs were frequently observed within individual host cells infected with the *cig2::*Tn mutant, and these CCVs were generally smaller than those formed by wild-type bacteria (Fig. 5, Table 2 and 3) (15, 16). The reduced CCV size in the *cig2::*Tn mutant infection correlated with markedly lower bacterial burdens: a total of 836 bacterial cells were segmented across mutant CCVs (Table 2; Movie S2), compared to 3,306 cells within the four CCVs segmented in the wild-type data set at the same time point (Table 3; Movie S3). Several *cig2::*Tn mutant CCVs contained only a single bacterium, and 11 out of 16 CCVs contained three or fewer bacteria (Fig. 5 and Table 2; Movie S2).

**TABLE 2:** Statistics of the *cig2*::Tn mutant at 8 dpi

CCV	SCV	LCV	Intermediate	Total cells	Volume (µm^3^)
1	0	2	0	2	0.2
2	0	1	0	1	0.3
3	0	0	0	1	0.3
4	0	1	0	1	0.4
5	0	1	0	1	0.5
6	0	1	0	1	0.6
7	0	1	0	1	0.9
8	0	1	0	1	2.9
9	0	7	0	7	3.4
10	1	2	2	5	3.6
11	10	7	0	17	4.1
12	1	3	1	5	5.4
13	1	9	0	10	19.3
14	0	12	1	13	27.4
15	19	46	5	70	89.3
16	438	199	63	700	183.7
Average	29.4	18.3	4.6	52.3	20.2
Total	470	293	73	836	342.8

**TABLE 3:** Statistics of wild-type *C. burnetii* at 8 dpi

CCV	SCV	LCV	Intermediate	Total cells	Volume (µm^3^)
1	9	8	0	17	5.5
2	816	287	52	1,155	184.1
3	99	245	2	346	239
4	1,062	605	121	1,788	324.7
Average	496.5	286.3	43.8	826.5	188.3
Total	1,986	1,145	175	3,306	753.3

Despite these differences in CCV organization and bacterial load, both wild-type and *cig2::*Tn mutant populations exhibited pronounced bacterial pleiomorphism at 8 dpi. However, the ranges of bacterial cell volumes differed substantially between the two strains. In the *cig2::*Tn mutant infection, nearly all SCVs and intermediate cells were smaller than 20,000 voxels (~0.034 µm³), while LCVs clustered just below 40,000 voxels (~0.068 µm³) and extended beyond 100,000 voxels (~0.2 µm³) at the upper extreme (Fig. 5D and E). By contrast, wild-type bacteria at 8 dpi were significantly smaller overall (*P* < 0.0001). Most wild-type SCVs and intermediate cells were below 5,000 voxels (~0.009 µm³), and most LCVs were below 20,000 voxels (~0.034 µm³), with only a few cells exceeding 40,000 voxels (~0.068 µm³) (Fig. 6B, C and E).

Interestingly, despite these striking size differences, wild-type and *cig2::*Tn mutant populations displayed similar proportions of SCVs, LCVs, and intermediate cells at 8 dpi (Fig. 6F). By contrast, in the persistent infection FIB-SEM data set, wild-type *C. burnetii* exhibited a modestly higher SCV-to-LCV ratio but significantly larger average cell volumes compared to the 8 dpi time point (Fig. 6E and F). Taken together, these findings demonstrate that *C. burnetii* pleiomorphism is also observed at 8 dpi and is strongly influenced by CCV biogenesis, bacterial burden, and infection stage.

## DISCUSSION

FIB-SEM is emerging as a powerful approach for studying infections caused by intracellular pathogens at unprecedented resolution across whole-cell volumes (24–28). In this study, we combined FIB-SEM with machine learning-based quantitative analyses to visualize *C. burnetii* infection in three dimensions at a scale and resolution not previously achievable. This integrative workflow provides a substantially more comprehensive view of the infection process than earlier approaches and can be readily adapted to diverse host-pathogen systems and biological questions.

The CCV is a specialized organelle that matures along the endocytic pathway and is extensively remodeled by Dot/Icm T4SS effector proteins to support bacterial replication (5, 9, 14, 21). One such effector, Cig2, promotes fusion of the nascent CCV with autophagosomes and is required for the formation of the large, highly fusogenic vacuoles characteristic of wild-type infection (14–16). Consistent with previous reports, our FIB-SEM analysis revealed that *cig2::*Tn mutant-infected cells at 8 dpi exhibited a multivacuolar phenotype, with CCVs that were markedly smaller than those formed by wild-type bacteria at the same time point. Notably, bacterial cells within these smaller CCVs were significantly larger on average than their wild-type counterparts (Fig. 5 and 6). One possibility is that disruption of homotypic CCV fusion alters nutrient availability, spatial constraints, or signaling cues within the vacuole, thereby affecting regulation of bacterial growth and morphology. Further work will be required to dissect how CCV architecture feeds back onto bacterial physiology.

Interactions between intracellular pathogens and host cell organelles play critical roles in many infection systems. For example, host mitochondria associate with the *Salmonella*-containing vacuole as part of a host defense response (29), while *Legionella pneumophila* extensively remodels the ER around its vacuole via T4SS effector activity (30). By contrast, our analysis revealed relatively few direct contacts between host cell organelles and CCVs. Nevertheless, we observed short, linear densities apparently linking individual *C. burnetii* cells to host cell mitochondria and the ER (Fig. 4). Whether these structures represent functional tethers, transient membrane remnants from fusion events, or imaging artifacts cannot be resolved from the present data and will require targeted investigation using complementary approaches.

*C. burnetii* undergoes a distinctive biphasic developmental cycle, alternating between the infectious SCV and the replicative LCV (7, 8, 10). While SCV-to-LCV differentiation is triggered by acidification of the phagolysosome during endocytic maturation, the signal governing LCV-to-SCV differentiation remains unknown (7). Using the three-dimensional quantitative framework, we measured, for the first time, the distribution of bacterial variants across multiple CCVs within infected cells. The proportion of SCVs and LCVs varied widely among CCVs, even within the same infection condition. Although no strong correlation was observed between SCV abundance and CCV size, the two largest CCVs in the persistent wild-type infection FIB-SEM data set also exhibited the highest proportions of SCVs (Fig. 3), suggesting that these vacuoles represent late stages of infection. Definitive testing of this hypothesis will require more synchronized infection experiments that capture population dynamics and CCV maturation across the infection time course.

Importantly, our data indicate that bacterial pleiomorphism is not tightly linked to time post-infection. CCVs analyzed at 8 dpi—presumably at similar stages of maturation—displayed striking variability in bacterial size distributions and variant composition (Fig. 5D and E and 6B and C). One of the most intriguing observations was that in persistently infected cells, the largest SCVs exceeded the size of the largest LCVs, whereas at 8 dpi, SCVs were consistently smaller than LCVs (Fig. 3C,5D and 6B). These unusually large SCVs may represent cells that have not yet undergone the membrane contraction previously observed by cryo-ET (7), highlighting the dynamic and potentially reversible nature of *C. burnetii* developmental states.

In summary, this study establishes an advanced, scalable workflow that integrates FIB-SEM with machine learning-based quantitative analysis to visualize and characterize *C. burnetii* infection in three dimensions across large cellular volumes. By revealing extensive pleiomorphism in both bacterial cells and CCVs and uncovering unexpected relationships among vacuole architecture, bacterial morphology, and infection stage, our approach opens new avenues for dissecting the cellular and molecular mechanisms underlying *C. burnetii* pathogenesis and provides a broadly applicable framework for studying other intracellular pathogens.

## MATERIALS AND METHODS

### Bacterial strains, cell lines, and growth conditions

*C. burnetii* Nine Mile RSA439 (phase II, clone 4) (NMII) (31) and a *cig2* transposon insertion mutant (*cig2*::Tn) (14) were cultured axenically in liquid acidified citrate cysteine medium 2 (ACCM-2; Sunrise Science Products) for 6–8 days at 37°C, 5% CO_2_, and 2.5% O_2_ as previously described (32, 33). When appropriate, chloramphenicol (3 μg/mL) was added to ACCM-2. To calculate multiplicities of infection (MOIs), *C. burnetii* genome equivalents were enumerated by quantitative PCR using *dotA*-specific primers as previously described (14).

HeLa 229 cells (ATCC CCL-2.1) were grown in Dulbecco’s modified Eagle’s medium supplemented with 10% heat-inactivated fetal bovine serum at 37°C in 5% CO_2_.

### *C. burnetii* infections

For the persistent infection, HeLa cells were infected at an MOI of 500 with wild-type *C. burnetii* NMII. Infected cells were passaged by trypsinization and diluted 1:3 approximately every 3 days for about 2 months prior to fixation for microscopy. For the samples fixed at 8 dpi, HeLa cells were infected with either the wild-type *C. burnetii* NMII or a *cig2*::Tn mutant at a high MOI of 100–200. Infected cells were first expanded by trypsinization and then diluted approximately 1:2 once prior to fixation for microscopy.

### FIB-SEM

Infected cells were fixed in 2.5% glutaraldehyde and 2% paraformaldehyde prepared in 0.1 M sodium cacodylate buffer (pH 7.4) containing 2% sucrose for 1 h at room temperature. Samples were then rinsed in cacodylate buffer and incubated in 0.1% tannic acid in the same buffer for an additional hour. Post-fixation was performed in 1% osmium tetroxide and 1.5% potassium ferrocyanide in cacodylate buffer for 1 h. Samples were subsequently rinsed in cacodylate buffer followed by distilled water and en bloc stained overnight in 0.5% aqueous uranyl acetate. After extensive rinsing in distilled water, samples were incubated in lead aspartate at 60°C for 1 h, followed by a final rinse in distilled water.

Samples were dehydrated through a graded ethanol series to 100% ethanol and infiltrated with Embed 812 resin (Electron Microscopy Sciences). Resin-infiltrated samples were placed in silicone molds and polymerized at 60°C for at least 24 h. Resin blocks were trimmed to the approximate region of interest (ROI), and the block surface was freshly cut. The trimmed pyramid was carefully removed using a fine blade and mounted onto an aluminum stub using conductive carbon adhesive and silver paint (Electron Microscopy Sciences). To minimize charging during imaging, samples were sputter-coated with approximately 20 nm of platinum-palladium (Pt/Pd, 80:20) using a Cressington HR sputter coater (Ted Pella, Inc., Redding, CA, USA).

FIB-SEM imaging was performed using a dual-beam FIB-SEM (ZEISS Crossbeam 550) equipped with a gallium ion source. SmartSEM software (ZEISS, Jena, Germany) was used to identify ROIs and optimize imaging parameters based on SEM images acquired at 10 kV. Volumes measuring approximately 50 μm × 30 µm × 30 µm (width × height × depth) were acquired with an isotropic voxel size of 7 nm.

A protective platinum layer was deposited over the ROI using the FIB at 30 kV and 50 pA to protect surface structures and reduce charging. Carbon deposition, trench milling, and highlighting were performed at 30 kV and 3 nA, followed by coarse trench milling at 30 kV and 30 nA. Fine milling was carried out at 30 kV and 3 nA, and final serial sectioning of the imaging volume was performed at 30 kV and 300 pA.

After removal of each slice, images were acquired using a backscattered electron detector at an accelerating voltage of 1.5 kV, an imaging current of 2 nA, and an aperture diameter of 100 μm, with a pixel dwell time of 2 μs. Preliminary alignment of the image stacks was performed using Atlas 5 software (ZEISS), and aligned data sets were exported in TIFF format for downstream analysis.

### Segmentation, visualization, and data analysis

The FIB-SEM images were imported and analyzed using Dragonfly software (Comet Technologies Canada Inc., Montreal, Canada; https://www.theobjects.com/dragonfly [34]). Automatic data segmentation was achieved using CNNs with a U-net architecture. Ground truth was generated by manual segmentation of 5–10 slices before models were trained. Models trained to segment bacteria were applied only to volumes annotated as CCV, while models to segment mitochondria were applied to whole data sets. It was necessary to train separate models for each data set. Minimal postprocessing was used to correct automatic segmentations. Volumes and positions of annotated entities were quantified using the connected components analysis feature within Dragonfly. Dragonfly was also used to generate all images of segmented data. Statistical analyses were performed in Prism 10.

## SUPPLEMENTAL MATERIAL

Fig. S1 iai.00007-26-s0001.docx Training and validation of a model to differentiate SCVs and LCVs.

Supplemental material iai.00007-26-s0002.docx Legends for supplemental movies.

Movie S1 iai.00007-26-s0003.mp4 Animation of FIB-SEM data from HeLa cells persistently infected with wild-type *C. burnetii* for approximately 2 months.

Movie S2 iai.00007-26-s0004.mp4 Animation of FIB-SEM data from HeLa cells infected with the *cig2*::Tn *C. burnetii* at 8 days post-infection.

Movie S3 iai.00007-26-s0005.mp4 Animation of FIB-SEM data from HeLa cells infected with wild-type *C. burnetii* at 8 days post-infection.

ASM does not own the copyrights to Supplemental Material that may be linked to, or accessed through, an article. The authors have granted ASM a non-exclusive, world-wide license to publish the Supplemental Material files. Please contact the corresponding author directly for reuse.

## References

[1] Eldin C, Mélenotte C, Mediannikov O, Ghigo E, Million M, Edouard S, Mege J-L, Maurin M, Raoult D. 2017. From Q fever to Coxiella burnetii infection: a paradigm change. Clin Microbiol Rev 30:115–190. doi:10.1128/CMR.00045-16 27856520 PMC5217791

[2] Moos A, Hackstadt T. 1987. Comparative virulence of intra- and interstrain lipopolysaccharide variants of Coxiella burnetii in the guinea pig model. Infect Immun 55:1144–1150. doi:10.1128/iai.55.5.1144-1150.1987 3570458 PMC260482

[3] Shaw EI, Voth DE. 2019. Coxiella burnetii: a pathogenic intracellular acidophile. Microbiology (Reading) 165:1–3. doi:10.1099/mic.0.000707 30422108 PMC6600347

[4] McCaul TF, Williams JC. 1981. Developmental cycle of Coxiella burnetii: structure and morphogenesis of vegetative and sporogenic differentiations. J Bacteriol 147:1063–1076. doi:10.1128/jb.147.3.1063-1076.1981 7275931 PMC216147

[5] Berón W, Gutierrez MG, Rabinovitch M, Colombo MI. 2002. Coxiella burnetii localizes in a Rab7-labeled compartment with autophagic characteristics. Infect Immun 70:5816–5821. doi:10.1128/IAI.70.10.5816-5821.2002 12228312 PMC128334

[6] Burette M, Bonazzi M. 2020. From neglected to dissected: how technological advances are leading the way to the study of Coxiella burnetii pathogenesis. Cell Microbiol 22:e13180. doi:10.1111/cmi.13180 32185905

[7] Park D, Steiner S, Shao M, Roy CR, Liu J. 2022. Developmental transitions coordinate assembly of the Coxiella burnetii Dot/Icm type IV secretion system. Infect Immun 90. doi:10.1128/iai.00410-22 PMC9584302 36190257

[8] Coleman SA, Fischer ER, Howe D, Mead DJ, Heinzen RA. 2004. Temporal analysis of Coxiella burnetii morphological differentiation. J Bacteriol 186:7344–7352. doi:10.1128/JB.186.21.7344-7352.2004 15489446 PMC523218

[9] Howe Dale, Melnicákova J, Barák I, Heinzen RA. 2003. Fusogenicity of the Coxiella burnetii parasitophorous vacuole. Ann N Y Acad Sci 990:556–562. doi:10.1111/j.1749-6632.2003.tb07426.x 12860689

[10] Howe D, Mallavia LP. 2000. Coxiella burnetii exhibits morphological change and delays phagolysosomal fusion after internalization by J774A.1 cells. Infect Immun 68:3815–3821. doi:10.1128/IAI.68.7.3815-3821.2000 10858189 PMC101653

[11] Newton HJ, McDonough JA, Roy CR. 2013. Effector protein translocation by the Coxiella burnetii Dot/Icm type IV secretion system requires endocytic maturation of the pathogen-occupied vacuole. PLoS One 8:e54566. doi:10.1371/journal.pone.0054566 23349930 PMC3547880

[12] Newton P, Thomas DR, Reed SCO, Lau N, Xu B, Ong SY, Pasricha S, Madhamshettiwar PB, Edgington-Mitchell LE, Simpson KJ, Roy CR, Newton HJ. 2020. Lysosomal degradation products induce Coxiella burnetii virulence. Proc Natl Acad Sci USA 117:6801–6810. doi:10.1073/pnas.1921344117 32152125 PMC7104363

[13] Thomas DR, Newton P, Lau N, Newton HJ. 2020. Interfering with autophagy: the opposing strategies deployed by Legionella pneumophila and Coxiella burnetii effector proteins. Front Cell Infect Microbiol 10:599762. doi:10.3389/fcimb.2020.599762 33251162 PMC7676224

[14] Newton HJ, Kohler LJ, McDonough JA, Temoche-Diaz M, Crabill E, Hartland EL, Roy CR. 2014. A screen of Coxiella burnetii mutants reveals important roles for Dot/Icm effectors and host autophagy in vacuole biogenesis. PLoS Pathog 10:e1004286. doi:10.1371/journal.ppat.1004286 25080348 PMC4117601

[15] Kohler LJ, Reed SCO, Sarraf SA, Arteaga DD, Newton HJ, Roy CR. 2016. Effector protein Cig2 decreases host tolerance of infection by directing constitutive fusion of autophagosomes with the Coxiella-containing vacuole. mBio 7:e01127-16. doi:10.1128/mBio.01127-16 27435465 PMC4958265

[16] Martinez E, Allombert J, Cantet F, Lakhani A, Yandrapalli N, Neyret A, Norville IH, Favard C, Muriaux D, Bonazzi M. 2016. Coxiella burnetii effector CvpB modulates phosphoinositide metabolism for optimal vacuole development. Proc Natl Acad Sci USA 113:E3260–9. doi:10.1073/pnas.1522811113 27226300 PMC4988616

[17] Latomanski EA, Newton P, Khoo CA, Newton HJ. 2016. The effector Cig57 hijacks FCHO-mediated vesicular trafficking to facilitate intracellular replication of Coxiella burnetii. PLoS Pathog 12:e1006101. doi:10.1371/journal.ppat.1006101 28002452 PMC5176192

[18] Latomanski EA, Newton HJ. 2018. Interaction between autophagic vesicles and the Coxiella-containing vacuole requires CLTC (clathrin heavy chain). Autophagy 14:1710–1725. doi:10.1080/15548627.2018.1483806 29973118 PMC6135622

[19] Beare PA, Gilk SD, Larson CL, Hill J, Stead CM, Omsland A, Cockrell DC, Howe D, Voth DE, Heinzen RA. 2011. Dot/Icm type IVB secretion system requirements for Coxiella burnetii growth in human macrophages. mBio 2:e00175-11. doi:10.1128/mBio.00175-11 21862628 PMC3163939

[20] Carey KL, Newton HJ, Lührmann A, Roy CR. 2011. The Coxiella burnetii Dot/Icm system delivers a unique repertoire of type IV effectors into host cells and is required for intracellular replication. PLoS Pathog 7:e1002056. doi:10.1371/journal.ppat.1002056 21637816 PMC3102713

[21] Kohler LJ, Roy CR. 2015. Biogenesis of the lysosome-derived vacuole containing Coxiella burnetii. Microbes Infect 17:766–771. doi:10.1016/j.micinf.2015.08.006 26327296 PMC4666725

[22] McDonough JA, Newton HJ, Klum S, Swiss R, Agaisse H, Roy CR. 2013. Host pathways important for Coxiella burnetii infection revealed by genome-wide RNA interference screening. mBio 4:e00606-12. doi:10.1128/mBio.00606-12 23362322 PMC3560531

[23] Weiner A, Enninga J. 2019. The pathogen-host interface in three dimensions: correlative FIB/SEM applications. Trends Microbiol 27:426–439. doi:10.1016/j.tim.2018.11.011 30600140

[24] Lê-Bury G, Deschamps C, Kizilyaprak C, Blanchard W, Daraspe J, Dumas A, Gordon MA, Hinton JCD, Humbel BM, Niedergang F. 2020. Increased intracellular survival of Salmonella Typhimurium ST313 in HIV-1-infected primary human macrophages is not associated with Salmonella hijacking the HIV compartment. Biol Cell 112:92–101. doi:10.1111/boc.201900055 31922615

[25] Regmi KC, Ghosh S, Koch B, Neumann U, Stein B, O’Connell RJ, Innes RW. 2024. Three-dimensional ultrastructure of arabidopsis cotyledons infected with Colletotrichum higginsianum. Mol Plant Microbe Interact 37:396–406. doi:10.1094/MPMI-05-23-0068-R 38148303

[26] Antao NV, Lam C, Davydov A, Riggi M, Sall J, Petzold C, Liang FX, Iwasa JH, Ekiert DC, Bhabha G. 2023. 3D reconstructions of parasite development and the intracellular niche of the microsporidian pathogen Encephalitozoon intestinalis. Nat Commun 14:7662. doi:10.1038/s41467-023-43215-0 37996434 PMC10667486

[27] Acevedo-Sánchez Y, Woida PJ, Kraemer S, Lamason RL. 2023. An obligate intracellular bacterial pathogen forms a direct, interkingdom membrane contact site. bioRxiv:2023.06.05.543771. doi:10.1101/2023.06.05.543771

[28] Klose M, Scheungrab M, Luckner M, Wanner G, Linder S. 2021. FIB-SEM-based analysis of Borrelia intracellular processing by human macrophages. J Cell Sci 134:jcs252320. doi:10.1242/jcs.252320 33380490

[29] Lian H, Park D, Chen M, Schueder F, Lara-Tejero M, Liu J, Galán JE. 2023. Parkinson’s disease kinase LRRK2 coordinates a cell-intrinsic itaconate-dependent defence pathway against intracellular Salmonella. Nat Microbiol 8:1880–1895. doi:10.1038/s41564-023-01459-y 37640963 PMC10962312

[30] Ragaz C, Pietsch H, Urwyler S, Tiaden A, Weber SS, Hilbi H. 2008. The Legionella pneumophila phosphatidylinositol-4 phosphate-binding type IV substrate SidC recruits endoplasmic reticulum vesicles to a replication-permissive vacuole. Cell Microbiol 10:2416–2433. doi:10.1111/j.1462-5822.2008.01219.x 18673369

[31] Williams JC, Peacock MG, McCaul TF. 1981. Immunological and biological characterization of Coxiella burnetii, phases I and II, separated from host components. Infect Immun 32:840–851. doi:10.1128/iai.32.2.840-851.1981 7251150 PMC351520

[32] Omsland A, Cockrell DC, Howe D, Fischer ER, Virtaneva K, Sturdevant DE, Porcella SF, Heinzen RA. 2009. Host cell-free growth of the Q fever bacterium Coxiella burnetii. Proc Natl Acad Sci USA 106:4430–4434. doi:10.1073/pnas.0812074106 19246385 PMC2657411

[33] Omsland A, Beare PA, Hill J, Cockrell DC, Howe D, Hansen B, Samuel JE, Heinzen RA. 2011. Isolation from animal tissue and genetic transformation of Coxiella burnetii are facilitated by an improved axenic growth medium. Appl Environ Microbiol 77:3720–3725. doi:10.1128/AEM.02826-10 21478315 PMC3127619

[34] Comet Technologies canada Inc. 2025 Dragonfly 3D World. Comet Technologies Inc., Montreal, Canada. Available from: https://dragonfly.comet.tech

