# Supplementary material for: Quantitative three-dimensional imaging of Coxiella burnetii infection by focused ion beam-scanning electron microscopy
Source: Infect Immun. 2026 Apr 29;94(6):e00007-26. doi: 10.1128/iai.00007-26 (PMC13248727; doi:10.1128/iai.00007-26)
Supplement: Fig. S1 — Training and validation of a model to differentiate SCVs and LCVs. [file iai.00007-26-s0001.docx]

**Supplemental Materials**

**
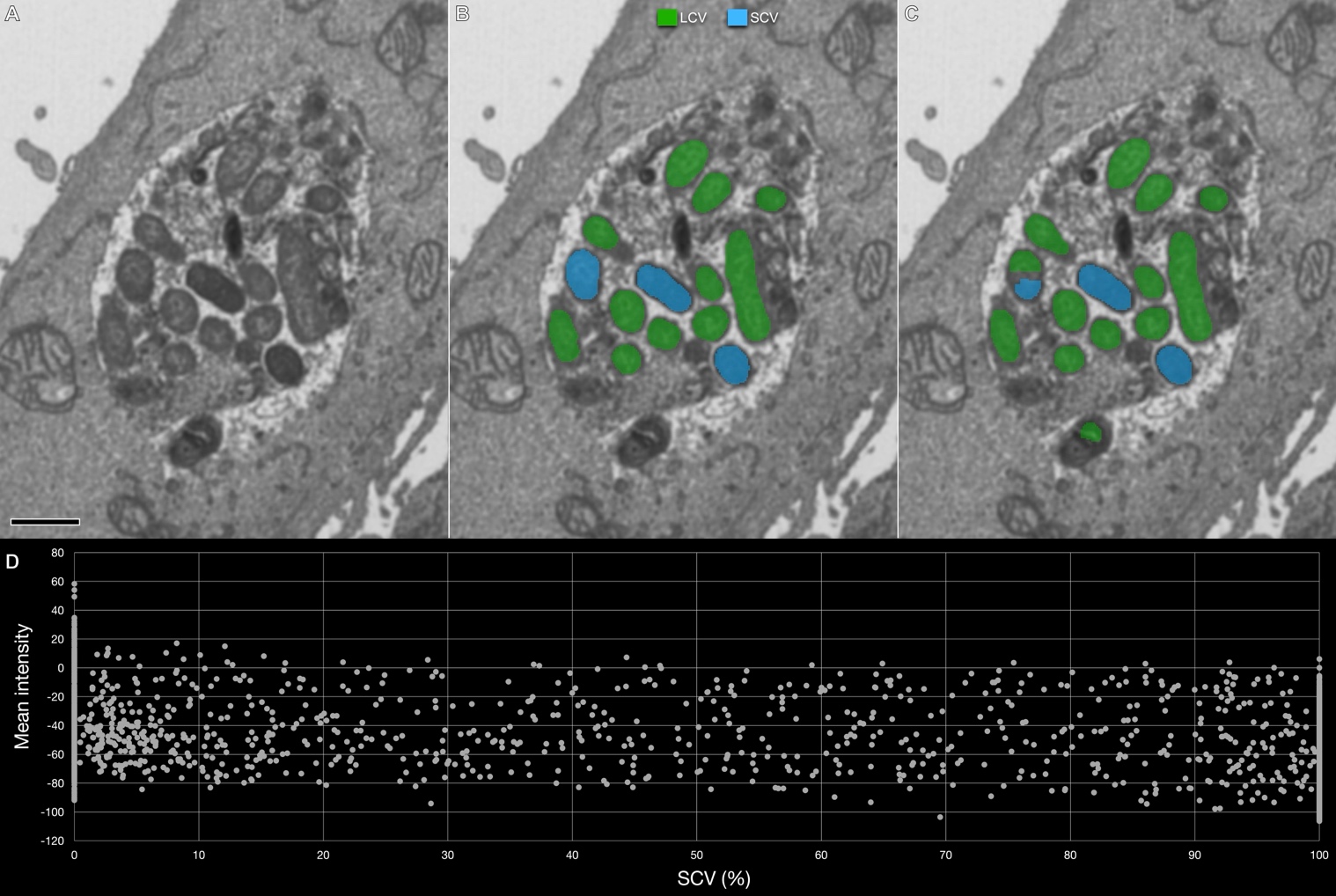
**

**Figure S1 | Training and validation of a model to differentiate SCVs and LCVs.** (**A**) Representative slice from the FIB-SEM dataset showing a CCV. Scale bar, 1 μm. (**B**) Pseudocolored annotation of the slice shown in (**A**) used as ground truth for model training, with *C. burnetii* cells manually classified as SCVs (blue) or LCVs (green). (**C**) Output of the trained model applied to the same slice, showing voxel-level classification of *C. burnetii* cells as SCVs (blue), LCVs (green), or neither (grayscale). (**D**) Scatter plot showing the relationship between mean electron density of individual bacterial cells and the proportion of voxels classified as SCV by the model, demonstrating that variant classification is not simply driven by average electron density.
