# Supplementary material for: Quantitative three-dimensional imaging of Coxiella burnetii infection by focused ion beam-scanning electron microscopy
Source: Infect Immun. 2026 Apr 29;94(6):e00007-26. doi: 10.1128/iai.00007-26 (PMC13248727; doi:10.1128/iai.00007-26)
Supplement: Supplemental material — Legends for supplemental movies. [file iai.00007-26-s0002.docx]

Movie S1

Animation of FIB-SEM data from HeLa cells persistently infected with wild-type C. burnetii for approximately 2 months.

Movie S2

Animation of FIB-SEM data from HeLa cells infected with the cig2::Tn C. burnetii at 8 days post-infection.

Movie S3

Animation of FIB-SEM data from HeLa cells infected with wild-type C. burnetii at 8 days post-infection.
